# Évaluation de l’efficacité et de la tolérance de l’acide tranexamique associé à l’antivenin Inoserp^TM^ PAN-AFRICA dans le traitement du syndrome hémorragique au cours de l’envenimation par *Echis ocellatus* au Bénin

**DOI:** 10.48327/mtsi.v6i1.2026.745

**Published:** 2026-03-15

**Authors:** Sébastien LARRÉCHÉ, Noé SODJINOU, Naryanan TOURITA, Seidou Alassane OUOROU, Éric GANHOUINGNON, Édith ALOUKOUTOU LAYO, Achille MASSOUGBODJI, Jean-Philippe CHIPPAUX

**Affiliations:** 1 Service de biologie médicale, Hôpital national d’instruction des armées Bégin, 69 avenue de Paris, 94160 Saint-Mandé; École du Val-de-Grâce, 1 place Alphonse Laveran, 75005 Paris; UMR-S1144, Université Paris Cité, 4 avenue de l’observatoire, 75006, Paris, France; 2 Institut de recherche clinique du Bénin, Abomey-Calavi, Bénin; 3 Département Santé, Université Senghor, 1, Place Ahmed Orabi, Al Mancheya, BP 415, 21111 Alexandrie, Égypte; 4 Service des urgences, Hôpital Saint-Jean de Dieu, Tanguiéta, Bénin; 5 Laboratoire de biologie, Hôpital Saint-Jean de Dieu, Tanguiéta, Bénin; 6 Université Paris Cité, IRD, Inserm, MERIT, F-75006 Paris, France

**Keywords:** Morsure de serpent, Viperidae, Coagulopathie, Fibrinolyse, Antifibrinolytique, Antivenin, Sérum antivenimeux, Bénin, Afrique subsaharienne, Snakebites, Viperidae, Coagulopathy, Fibrinolysis, Antifibrinolytic, Antivenom, Benin, Sub-Saharan Africa

## Abstract

**Introduction:**

Le syndrome hémorragique contribue à une grande partie de la mortalité et de la morbidité associées aux morsures de serpent en Afrique subsaharienne. L’hyperfibrinolyse rapportée dans certains cas suggère l’intérêt des antifibrinolytiques mais ces derniers ont été peu étudiés dans ce contexte. L’objectif de cette étude était d’évaluer l’intérêt de l’association de l’acide tranexamique et d’un antivenin dans le traitement du syndrome hémorragique et de la coagulopathie associés à l’envenimation par *Echis ocellatus* (échide ocellée).

**Matériel et méthode:**

Des patients envenimés présentant un syndrome hémorragique ont été inclus prospectivement à l’hôpital Saint Jean de Dieu de Tanguiéta, au Bénin. Les patients ont été randomisés en deux groupes : un groupe recevant un antivenin et de l’acide tranexamique (groupe SAV + AT) versus un groupe recevant uniquement l’antivenin (groupe SAV). Tous les patients ont reçu deux flacons d’antivenin Inoserp^TM^ PAN-AFRICA à leur admission. Dans le groupe SAV + AT, les patients ont reçu 1 g d’acide tranexamique en 30 minutes, puis 1 g en 8 heures. La survenue d’un saignement et le résultat du test de coagulation sanguine (test de coagulation sur tube sec ou TCTS) ont été recueillis 2, 4, 6, 8, 12, 24, 48 et 72 heures après la première administration d’antivenin. L’administration d’antivenin était renouvelée en cas de saignement à l’un de ces temps. L’INR (*International Normalized Ratio*) a été mesuré à 6 et 12 heures. La tolérance a été évaluée à 1, 2, 4, 6, 8, 12, 24, 48 et 72 heures. La créatinine plasmatique a été mesurée à 24 et 72 heures.

**Résultats:**

Vingt-cinq patients ont été inclus : 12 patients dans le groupe SAV + AT contre 13 patients dans le groupe SAV. Six patients ont présenté une persistance ou une récidive du saignement dans le groupe SAV + AT contre 5 dans le groupe SAV (p = 0,695). Aucune différence significative du TCTS n’a été observée aux différents temps entre les deux groupes, ni pour l’INR à 6 et 12 h. Aucun effet indésirable suggérant une intolérance à l’acide tranexamique n’a été constaté (anomalie oculaire, convulsions, thrombose), tandis que la créatinine plasmatique était sans différence significative entre les deux groupes.

**Conclusion:**

L’utilisation systématique de l’acide tranexamique n’a montré aucun bénéfice dans le syndrome hémorragique et la coagulopathie associés à *E. ocellatus* au Bénin. Les indications de ce traitement d’appoint méritent donc d’être précisées.

## Abstract (en)

**Introduction:**

Hemorrhagic syndrome accounts for a significant proportion of the mortality and morbidity associated with snakebites in sub-Saharan Africa. Hyperfibrinolysis, which has been reported in some cases, suggests the potential benefit of antifibrinolytics; however, these have been little studied in this context. This study aimed to evaluate the effectiveness of combining tranexamic acid and antivenom in treating hemorrhagic syndrome and coagulopathy associated with *Echis ocellatus* (West African Carpet Viper) envenomation.

**Materials and methods:**

Patients presenting with hemorrhagic syndrome due to envenomation were prospectively included at the Saint Jean de Dieu Hospital in Tanguiéta, Benin. Patients were randomized into two groups: one receiving antivenom and tranexamic acid (SAV + AT) and one receiving only antivenom (SAV). All patients received two vials of Inoserp^TM^ PAN-AFRICA antivenom upon admission. The SAV + AT group received 1 g of tranexamic acid over 30 minutes, followed by an additional 1 g over 8 hours. The occurrence of bleeding and the results of blood coagulation tests (whole blood clotting test or WBCT) were recorded at 2, 4, 6, 8, 12, 24, 48, and 72 hours after the initial antivenom administration. Antivenom administration was repeated if bleeding occurred at any of these times. The INR (International Normalized Ratio) was measured at six and twelve hours. Tolerance was assessed at 1, 2, 4, 6, 8, 12, 24, 48, and 72 hours. Plasma creatinine was measured at 24 and 72 hours.

**Results:**

Twenty-five patients were included: Twelve patients were in the SAV + AT group, and thirteen patients were in the SAV group. Six patients in the SAV + AT group and five patients in the SAV group experienced persistent or recurrent bleeding (p = 0.695). There was no significant difference in WBCT at different time points between the two groups, nor in INR at 6 and 12 hours. There were no adverse effects suggesting intolerance to tranexamic acid (e.g., ocular abnormality, convulsions, or thrombosis), and plasma creatinine levels did not differ significantly between the two groups.

**Conclusion:**

Systematic use of tranexamic acid showed no benefit for hemorrhagic syndrome and coagulopathy associated with *E. ocellatus* in Benin. Therefore, the indications for this adjunctive treatment need to be clarified.

**Figure:**
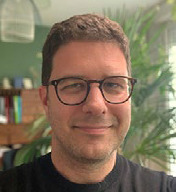

## Introduction

En Afrique subsaharienne, l’incidence et la gravité des morsures de serpent constituent un problème de santé publique négligé, avec plus de 300 000 envenimations traitées dans les centres de santé chaque année [7]. Un syndrome hémorragique participe pour une grande partie à la mortalité et à la morbidité associées à ces envenimations en savane soudanienne dont fait partie le nord du Bénin. Dans cette région africaine, l’espèce la plus souvent incriminée est *Echis ocellatus* (échide ocellée), un vipéridé de petite taille mais au puissant venin. La morsure d’*E. ocellatus* est marquée par un saignement local persistant. Dans certains cas, des manifestations hémorragiques systémiques peuvent survenir : phlyctènes, saignement des muqueuses, hémorragie digestive, hémorragie cérébrale ou méningée. Ces atteintes peuvent alors conduire au décès du patient [39]. Ces saignements sont le fait d’une physiopathologie complexe associant destruction vasculaire, atteinte plaquettaire et coagulopathie de consommation. Cette dernière est principalement due à la présence d’activateurs de la prothrombine, entraînant sa transformation en thrombine, aboutissant à la quasi-disparition du fibrinogène [16,34].

Le traitement de l’envenimation ophidienne, et donc du syndrome hémorragique induit par le venin, repose sur l’antivenin. Différents travaux ont montré une efficacité sur les saignements et sur la coagulopathie de consommation [10,11,24]. Cependant, chez certains patients, une persistance ou une récidive du saignement a été rapportée sous traitement. La recommandation actuelle est alors d’administrer une nouvelle dose d’antivenin. Outre le risque de majoration des effets indésirables [21], cette attitude augmente le coût du traitement, généralement à la charge du patient. Au Bénin, le coût médian de l’antivenin nécessaire pour une envenimation est supérieur au revenu mensuel moyen par habitant [38]. Il est donc nécessaire d’évaluer l’intérêt d’autres traitements moins coûteux. Parmi eux, l’acide tranexamique est fréquemment utilisé dans les centres de santé prenant en charge ces envenimations en Afrique subsaharienne. Il s’agit d’un antifibrinolytique peu onéreux qui inhibe la formation de plasmine, par blocage des sites de liaison de la lysine au niveau du plasminogène. Toutefois, peu de données cliniques sont disponibles sur son utilisation dans le cadre des envenimations par morsure de serpent. L’objectif de cette étude était d’évaluer l’efficacité et la tolérance de l’association acide tranexamique et antivenin dans le traitement du syndrome hémorragique et de la coagulopathie associée à l’envenimation par *E. ocellatus* au Bénin.

## Matériel et méthodes

Cet essai clinique contrôlé randomisé de phase III s’est déroulé à l’hôpital Saint Jean de Dieu, à Tanguiéta, dans le nord-ouest du Bénin, du 30 mai au 29 octobre 2023. Les patients se présentant pour une morsure de serpent avec un saignement actif ont été inclus dans l’étude.

Outre le refus de participer à l’étude, les critères de non-inclusion étaient une morsure datant de plus de 4 jours, l’administration d’un antivenin préalable à l’arrivée à l’hôpital Saint-Jean de Dieu ou la présence d’une contre-indication à l’acide tranexamique (thrombose artérielle ou veineuse, insuffisance rénale sévère avec clairance de la créatinine < 30 ml/min, convulsions actuelles ou rapportées dans les antécédents, antécédent d’épilepsie, grossesse au 1^er^ trimestre ou allaitement, hypersensibilité connue à l’acide tranexamique. Les critères d’exclusion étaient la découverte d’une clairance de la créatinine < 30 ml/min sur le bilan biologique d’admission ou la décision du patient de se retirer de l’étude.

Les patients non inclus ont reçu le traitement standard recommandé par le ministère de la santé du Bénin.

Les patients inclus ont été randomisés en deux groupes à l’admission (H0) : un groupe recevant l’Inoserp^TM^ PAN-AFRICA et l’acide tranexamique (groupe SAV + AT) et un groupe recevant uniquement l’Inoserp^TM^ PAN-AFRICA (groupe SAV). L’inclusion d’un patient conduisait à l’ouverture d’une fiche de recueil sur le site EasyMedStat (version 3.27; www.easymedstat.com). Un numéro d’identification était généré automatiquement par le site pour chaque fiche : les patients avec un numéro pair étaient inclus dans le groupe SAV + AT et ceux avec un numéro impair dans le groupe SAV. Il est apparu que les numéros générés étaient systématiquement impairs, aussi la consigne d’inclusion a été inversée à partir du 16 août, alors que 13 patients étaient déjà inclus : un numéro pair faisait inclure dans le groupe SAV et un numéro impair dans le groupe SAV + AT. Malgré ce changement de règle de recrutement, les trois patients suivants ont été attribués au groupe SAV. Il a donc été décidé à partir du 30 août d’admettre systématiquement les nouveaux patients dans le groupe SAV + AT pour équilibrer les effectifs, soit 9 nouveaux patients recevant le SAV et l’AT.

L’Inoserp^TM^ PAN-AFRICA (Inosan Biopharma, Madrid, Espagne) est un antivenin polyvalent lyophilisé (lots 2IT02001 et 3IT01001). L’antivenin a été administré selon le protocole préconisé par la Société africaine de venimologie [8]. Chaque ampoule est à reconstituer avec 10 ml de solution saline injectable. Elle neutralise au moins 250 DL_50_ de venin d’*E. ocellatus*. La voie d’administration est la voie intraveineuse directe lente (injection en 5 minutes minimum). La posologie initiale est de deux ampoules. Une nouvelle évaluation clinique est réalisée 2 h (H2), 4 h (H4), 6 h (H6), 8 h (H8), 12 h (H12), 24 h (H24), 48 h (H48), 72 h (H72) après la 1^re^ administration d’antivenin : deux ampoules d’antivenin sont administrées à nouveau en cas de persistance ou de récidive d’un saignement. Une dernière évaluation était réalisée le jour du retour à domicile quelle que soit la durée de l’hospitalisation.

L’acide tranexamique (Exacyl, Kwality Pharmaceuticals Ltd, Amritsar, Inde, lot N-17207) est administré selon le protocole suivant : une dose de charge de 2 ampoules de 500 mg dans une perfusion de 250 ml de glucosé 5 % en 30 minutes puis une dose d’entretien de 2 ampoules de 500 mg dans une perfusion de 500 ml de NaCl 0,9 % en 8 heures par voie intraveineuse. Il était prévu de ne pas administrer de dose d’entretien en cas de créatininémie supérieure à 250 µmol/l sur le bilan réalisé à l’admission. Pour les enfants (patients âgés de moins de 18 ans), les doses de charge et d’entretien étaient de 10 mg/kg (1 g maximum). L’apparition de troubles de la vision, d’une anurie, d’une convulsion ou d’une thrombose devait faire arrêter la perfusion d’acide tranexamique. Pour les patients du groupe SAV, la perfusion suivante était prévue : 250 ml de glucosé 5 % en 30 minutes puis 500 ml de NaCl 0,9 % en 8 heures.

Un formulaire de recueil standardisé a été rempli avec les caractéristiques du patient : genre, âge, délai entre morsure et admission, identification du serpent, grade de l’œdème et de l’hémorragie (Tableau I), présence de nécrose ou d’autres signes cliniques, résultats biologiques, traitement (nombre d’ampoules d’antivenin et d’acide tranexamique, date et heure d’administration). Un œdème important était défini par un grade de l’œdème ≥ 2. Un saignement systémique était défini par un grade de l’hémorragie ≥ 2. Les serpents incriminés dans les morsures et rapportés à l’hôpital étaient identifiés par un herpétologue. Dans cette région du Bénin, la seule espèce responsable d’envenimation avec un syndrome hémorragique est *E. ocellatus*. Ainsi, même si le serpent n’a pu être apporté, nous avons considéré qu’il s’agissait d’*E. ocellatus* en cas de saignement détecté à l’admission. Ces données ont été ensuite saisies dans une base de données sécurisée en ligne sur le site EasyMedStat.

Le patient était ensuite surveillé pendant une heure puis aux différentes visites précisées cidessus pour rechercher un éventuel signe d’intolérance. Tous les symptômes ou signes apparaissant après l’administration ou la réadministration d’Inoserp^TM^ PAN-AFRICA ou d’acide tranexamique et évoquant une possible intolérance ont été considérés comme des effets indésirables.

À l’admission, après inclusion et avant administration de l’antivenin, un échantillon de sang total veineux était prélevé pour un test de coagulation sur tube sec (TCTS), une numération formule sanguine et un dosage de la créatinine plasmatique. Le TCTS a été réalisé selon la méthode décrite par Benjamin *et al*. [5]. Quelques millilitres de sang total veineux sont déposés dans un tube en verre propre, maintenu droit à température ambiante. Le TCTS était lu à 3 temps : 20, 30 et 60 minutes. Pour chaque temps de lecture, la qualité du caillot était appréciée. Un caillot normal reste fixé lorsque le tube est horizontalisé (grade 0). Si le sang reste liquide, la présence d’un petit caillot ou d’un caillot qui se détruit lors de la manipulation du tube est un grade 1. L’absence de caillot est un grade 2. Le TCTS est considéré comme anormal pour les caillots classés 1 ou 2 pour au moins un temps de lecture.

**Tableau I:** Critères de gradation de l’œdème et de l’hémorragie

Grade	Œdème	Hémorragie
0	Absence	Absence
1	N’excédant pas une articulation	Persistance d’un saignement au site de morsure pendant plus de 30 minutes
2	Atteignant deux articulations	Saignement des muqueuses ou des cicatrices récentes
3	Touchant l’ensemble du membre sans dépasser la racine	Saignement sous-cutané à distance de la morsure ou des cicatrices anciennes
4	Dépassant la racine	Externalisation d’un saignement interne ou saignement du système nerveux cérébral

Après administration de la 1^re^ dose d’antivenin, les patients ont été également prélevés à H2, H4, H6, H8, H12, H24, H48, H72 et le jour du retour à domicile. Un TCTS a été réalisé sur ces différents prélèvements, un INR à H6 et H12, un dosage de la créatinine plasmatique à H24 et H72. L’ajout de l’INR en plus du TCTS correspond à des recommandations récentes pour définir les critères de jugement d’un essai clinique sur les morsures de serpents : il est préconisé de mesurer ce paramètre à H6 et H12 pour évaluer l’efficacité d’un antivenin en cas d’envenimation avec coagulopathie [2].

Le critère de jugement principal était l’absence d’un saignement persistant ou récidivant. Un saignement persistant est défini comme un saignement encore présent à H2. Un saignement récidivant est défini comme un saignement absent à H2 puis présent à au moins une des évaluations suivantes au cours du suivi.

Les critères de jugement secondaires étaient la récidive d’une coagulopathie définie par un TCTS redevenant anormal après une correction notée à au moins une des évaluations précédentes au cours du suivi : l’INR à H6 et H12, le nombre total d’ampoules d’antivenin par patient, la présence d’effets indésirables, la valeur de la créatinine à H24 et H72.

L’analyse statistique a été réalisée avec GraphPad PRISM 9.5.0 (GraphPad Prism Inc., La Jolla, CA, États-Unis). Les variables qualitatives ont été exprimées en valeurs absolues et en pourcentages et les variables quantitatives sous forme de médiane, assortie des quartiles à 25 % et 75 %. La normalité des variables quantitatives a été vérifiée par le test de Shapiro-Wilk. Du fait de la faiblesse des effectifs, nous avons utilisé le test non paramétrique de Mann-Whitney pour comparer les médianes des variables quantitatives et le test exact de Fisher pour la comparaison des pourcentages. L’objectif de l’étude était de démontrer que l’association antivenin et acide tranexamique était plus efficace que la seule administration d’antivenin. Si les deux stratégies ont une efficacité similaire, l’ajout systématique ne serait pas opportun. Le test exact de Fisher a donc été réalisé en unilatéral pour comparer les groupes sur les critères de jugement. La différence statistique des résultats a été considérée comme significative lorsque p < 0,05.

Cet essai clinique a été approuvé par le Comité national d’éthique pour la recherche en santé (CNERS) du Bénin (Avis éthique favorable N°101/ MS/DC/SGM/CNERS/SA du 25 juillet 2022). Tous les participants (ou le cas échéant, leurs parents ou leurs tuteurs pour les mineurs) ont signé un consentement écrit après avoir reçu une information claire, appropriée et loyale.

## Résultats

Vingt-cinq patients ont été inclus : 12 dans le groupe SAV + AT et 13 dans le groupe SAV. Les données épidémiologiques, cliniques et biologiques à l’admission sont présentées dans le Tableau II. Aucune différence statistiquement significative n’a été mise en évidence pour ces différentes variables. Deux patients sont arrivés juste avant le délai limite de quatre jours. Aucune nécrose cutanée ou manifestation neurologique n’a été notée. Le serpent n’a été rapporté que pour un patient et a été identifié comme un *E. ocellatus*.

**Tableau II:** Description des caractéristiques des patients à l’admission

	Groupe SAV + ATN = 12	Groupe SAV = 13	p-Value
Sex-ratio homme/femme	8/4	10/3	0,673
Âge moyen (années)	23 (± 11,0)	29 (± 11)	0,207
Délai moyen morsure/admission (heures)	47,2 (± 31,4)	32,6 (± 28,8)	0,236
Œdème important	11 (92 %)	9 (69 %)	0,322
Saignement systémique	12 (100 %)	12 (92 %)	>0,999
TCTS anormal	12 (100 %)	13 (100 %)	>0,999
Hémoglobine (g/dl)	10,8 (± 4,2)	12,0 (± 3,7)	0,457
Plaquettes (G/l)	216 (± 95)	180 (± 56,6)	0,258
Créatinine (µmol/l)	84 (± 25)	93 (± 23)	0,383

TCTS : test de coagulation sur tube sec

**Figure 1:**
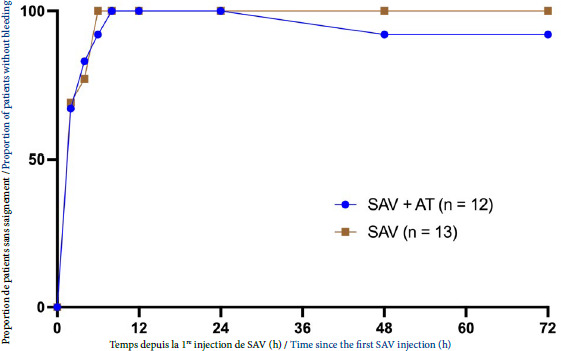
Correction du saignement au cours du temps

Le nombre de patients ayant corrigé leur saignement au cours du temps est présenté sur la Figure 1. Six patients (50 %) ont présenté un saignement persistant ou récidivant dans le groupe SAV + AT et 5 patients (38 %) dans le groupe SAV (p = 0,837). Quatre patients ont présenté un saignement persistant dans chaque groupe (p = 0,714). Les saignements persistants ont disparu chez l’ensemble des patients à partir de H8. Un saignement récidivant a été noté chez deux patients du groupe SAV + AT à H48 et à H72 et chez un patient du groupe SAV au-delà du suivi (au 4^e^ jour) (p = 0,904).

**Figure 2:**
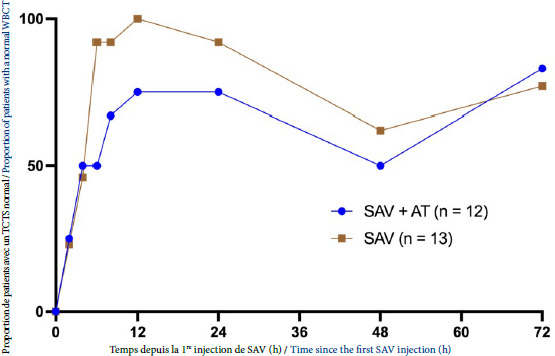
Correction du test de coagulation sur tube sec au cours du temps

Le nombre de patients ayant corrigé leur TCTS au cours du temps est présenté sur la Figure 2. Six patients (50 %) ont présenté une coagulopathie récidivante dans le groupe SAV + AT et 5 (38 %) patients dans le groupe SAV (p = 0,837).

**Figure 3:**
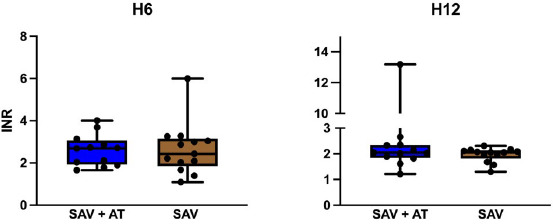
INR à la 6^e^ heure et à la 12^e^ heure après la 1^re^ administration d’antivenin

Aucune différence significative n’a été observée entre les deux groupes pour l’INR mesuré à H6 (p = 0,978) et à H12 (p = 0,663) (Fig. 3).

**Figure 4:**
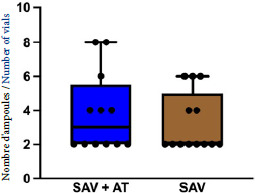
Nombre total d’ampoules d’antivenin par patient

Aucune différence significative n’a été observée entre les deux groupes pour le nombre total médian d’ampoules d’antivenin administrées par patient (p = 0,548) (Fig. 4).

Aucun décès n’a été observé au cours de l’étude. Dans le groupe SAV + AT, il est survenu un prurit chez deux patients, une urticaire généralisée chez un patient, des vertiges chez un patient et un malaise chez un patient. Dans le groupe SAV, il est survenu des vertiges chez deux patients et un malaise chez un patient. Aucun autre effet indésirable n’a été rapporté : en particulier, pas d’hypotension, pas de trouble de la vision, pas de convulsions, pas de thrombose. Aucune augmentation pathologique de la créatininémie n’a été notée dans le groupe SAV + AT et aucune différence significative n’a été observée avec le groupe SAV pour cette variable à H24 (79,27 ± 17,71 µmol/l *versus* 86,36 ± 18,47 µmol/l, p = 0,339) et à H72 (83,16 ± 20,86 µmol/l *versus* 83,1 ± 14,65 µmol/l, p = 0,993).

## Discussion

À notre connaissance, aucun travail ne s’est intéressé à l’association acide tranexamique et antivenin. Deux études prospectives ont évalué l’intérêt de l’acide tranexamique sans aucun antivenin administré en parallèle. La première étude réalisée en Irak ne retrouvait pas de différence significative pour la mortalité entre les patients présentant un saignement après morsure traité ou non par acide tranexamique, respectivement de 30 % de 20 patients et 48 % de 23 patients [1]. Il n’y avait pas de critères de jugement relatif au saignement ou à la coagulopathie. L’autre étude menée au Népal chez des patients envenimés par *Ahaetulla nasuta*, un Colubridae venimeux potentiellement responsable de syndrome hémorragique, a montré un bénéfice sur la correction de l’INR au 5^e^ et 6^e^ jour et sur la durée d’hospitalisation [32]. Chez ces patients, le traitement standard était l’association plasma frais congelé et vitamine K.

Plusieurs causes peuvent expliquer la persistance et la récidive des saignements. Hormis l’absence ou le retard de régénération des facteurs de la coagulation qui n’est pas corroboré par une insuffisance hépatique [23,24,30,39], la persistance ou la reprise du syndrome hémorragique pourraient être attribuées à deux facteurs : une fibrinolyse secondaire à l’activation toxinique de la coagulation ou la libération de venin résiduel à partir d’un foyer de séquestration hors d’atteinte de l’antivenin [9,19,20,28,30,33,35].

L’association antivenin et acide tranexamique visait à répondre aux deux causes évoquées de saignement. D’une part, la demi-vie des fragments d’anticorps de type F(ab’)_2_ de l’antivenin dans le compartiment plasmatique est d’une cinquantaine d’heures, ce qui devrait permettre de neutraliser le venin circulant pendant plusieurs jours [4,31]. D’autre part, l’acide tranexamique avait pour objectif de contrôler l’hyperfibrinolyse, surtout si celle-ci persistait après l’élimination du venin. Dans cet essai clinique portant sur le traitement du syndrome hémorragique et de la coagulopathie associés aux morsures d’*E. ocellatus*, l’association antivenin et acide tranexamique n’a pas fait la preuve de son efficacité.

Chez certains patients, l’antivenin ne semble pas empêcher la persistance ou la récidive des saignements. Ceci n’est pas spécifique de l’Inoserp ^TM^ Pan-Africa puisqu’également décrit avec d’autres antivenins tels que l’Antivipmyn^TM^ Africa (Bioclon, Mexico, Mexique) [10], l’EchiTAb Plus-ICP (Institut Clodomiro Picado, Vazquez de Coronado, Costa Rica) [12,37] et l’EchiTAb G (MicroPharm Ltd, Newcastle Emlyn, UK) [3,12]. Ce constat suggère que la coagulopathie récidivante est indépendante de l’antivenin utilisé et semble liée à un relargage de venin à distance qui n’est pas neutralisé par l’antivenin [33]. Les mécanismes sous-tendant cette libération secondaire restent méconnus.

Plusieurs hypothèses peuvent expliquer l’inefficacité de l’acide tranexamique dans notre étude. Tout d’abord, l’hyperfibrinolyse n’est pas constante au cours de l’envenimation par *E. ocellatus*. Seuls 26 % des patients étudiés en viscoélastométrie à Tanguiéta présentaient une hyperfibrinolyse [19]. Chez le traumatisé sévère, il a été démontré que l’acide tranexamique n’a d’intérêt dans la correction des troubles de l’hémostase que chez les patients présentant une hyperfibrinolyse [26]. Idéalement, il faudrait utiliser l’acide tranexamique uniquement chez les patients présentant cette atteinte biologique, mais ce diagnostic est le plus souvent impossible en routine. Une alternative consisterait à cibler des envenimations pour lesquelles l’hyperfibrinolyse est fréquente et constitue le principal trouble de l’hémostase. Le venin d’*Ahaetulla prasina*, une espèce voisine d’*Ahaetulla nasuta* dont le venin n’a pas été étudié à notre connaissance, possède une prédominance de métalloprotéinases de type III et une activité fibrinogénolytique [25]. Il est donc possible que le saignement observé au cours de l’envenimation par *A. nasuta* soit dû à une hyperfibrinolyse, ce qui expliquerait l’efficacité sur la correction de l’INR dans l’étude menée au Népal chez les patients envenimés par *A. nasuta* [32]. D’autres espèces ophidiennes sont responsables d’envenimations dominées par ce mécanisme physiopathologique. Par exemple, *Hypnale hypnale*, un vipéridé présent au Sri-Lanka et au sud de l’Inde, est doté d’un venin provoquant une coagulopathie marquée par une fibrinogénolyse isolée [29]. L’utilisation de l’acide tranexamique pourrait donc être étudiée dans les envenimations dues à cette espèce. La principale hypothèse expliquant l’échec de l’acide tranexamique semble être l’hypofibrinogénémie majeure observée au cours de l’envenimation par les espèces du genre *Echis* [24]. La consommation intense des facteurs de coagulation et en particulier du fibrinogène conduit à leur quasi-disparition. L’antivenin *per se* ne restaure pas les facteurs de coagulation tandis que leur synthèse hépatique nécessite un délai incompressible. La traduction de cet état en viscoélastométrie est un caillot très réduit pendant plusieurs jours malgré l’antivenin [19]. Une hyperfibrinolyse peut rendre ce caillot instable mais sa correction n’est pas suffisante pour assurer une hémostase efficace arrêtant le saignement. Dans un modèle porcin de traumatisme hépatique grave, il a été montré que l’acide tranexamique seul ne permet pas de restaurer l’hémostase dans les situations d’hypofibrinogénémie profonde pour lesquelles l’ajout de concentré de fibrinogène demeure indispensable [42]. Il pourrait être intéressant d’associer une transfusion de facteurs de coagulation afin de corriger plus rapidement ce déficit. Le concentré de fibrinogène est disqualifié du fait de son coût et de son manque de disponibilité dans les centres de santé périphériques amenés à prendre en charge la majorité des patients. L’alternative est la transfusion de plasma frais congelé qui contient au moins 2,5 g/l de fibrinogène [14] et qui est recommandé dans la coagulopathie intravasculaire disséminée avec manifestations hémorragiques [22]. En outre, il est peu coûteux, ce qui le rend compatible avec les moyens financiers de la plupart des victimes. Un essai clinique mené en Australie a montré que l’INR était corrigé plus rapidement chez les patients recevant du plasma frais congelé en complément de l’antivenin [17]. Un autre bénéfice de l’ajout du plasma frais congelé observé au Sri Lanka et en Inde est la réduction du nombre d’ampoules d’antivenin et ainsi du coût du traitement [15,18]. Cependant, la transfusion de plasma n’est pas anodine en Afrique et comporte un risque de transmission d’agents transmissibles par le sang tels que le VIH, le VHB et le VHC [40,41].

En contrepartie, nous avons observé que l’acide tranexamique présente une très bonne tolérance dans le contexte des morsures de serpent. En particulier, aucune complication thrombotique ou convulsive n’a été observée. Théoriquement, l’inhibition de la fibrinolyse pourrait conduire à une formation de caillots du fait de l’activation intense de la coagulation par le venin. En réalité, la consommation des facteurs de coagulation est responsable d’un phénotype hypocoagulant marqué qui protège les patients de ce risque thrombotique. Dans les études précédentes réalisées lors d’envenimations ophidiennes, la tolérance était également excellente [1,32]. De façon générale, l’acide tranexamique est bien toléré, notamment aux doses usuelles de 1 à 2 g/jour que nous avons utilisé. Les évènements thromboemboliques ne semblent pas augmenter de manière significative selon plusieurs méta-analyses [27,36]. Les convulsions sont un risque rare et dose-dépendant, surtout à dose élevée ou dans un contexte de chirurgie cardiaque [6,26].

Plusieurs limites de cette étude doivent être soulignées. Cette étude a un effectif modeste mais un plus grand nombre de patients n’aurait probablement pas permis de montrer une efficacité de l’acide tranexamique. En particulier, la plupart des saignements ne concernait que les muqueuses et il y avait peu de cas graves. De même, l’absence d’évènements indésirables peut être due à la taille de l’effectif. Les modifications des règles de recrutement dans les deux bras au cours de l’étude ont conduit à une absence de vraie randomisation. Toutefois, il est peu probable que cela ait eu un retentissement sur l’étude car il n’y a pas d’argument pour une modification des venins des serpents à cette échelle de temps et nous avons utilisé le même lot pour les différents médicaments tout au long de l’étude. Les morsures étant concentrées sur une période de l’année entre mai et octobre, nous avons arrêté l’inclusion fin octobre. L’analyse des données nous a amenés à ne pas poursuivre cette étude durant la saison des morsures suivante. L’excès de plasmine générée au cours de l’hyperfibrinolyse consomme le fibrinogène [13]. Il aurait été intéressant de mesurer ce paramètre, mais nous avons fait le choix d’utiliser des tests soit accessibles en routine tels que le TCTS, soit recommandés dans les essais cliniques portant sur les antivenins tels que l’INR.

## Conclusion

L’ajout systématique de l’acide tranexamique à l’antivenin ne semble pas avoir d’intérêt dans le traitement des saignements associés aux morsures d’*E. ocellatus* au Bénin. L’utilisation de ce traitement peu coûteux mérite toutefois d’être étudiée dans d’autres pays où les envenimations sont marquées par une hyperfibrinolyse fréquente et prédominante tels que les morsures d’*H. hypnale* dans le sous-continent indien.

## Remerciements

Nous remercions l’ensemble du personnel de l’hôpital Saint Jean de Dieu de Tanguiéta pour son accueil et son aide au cours de cette étude.

## Financement

Cette étude a été financée sur fonds propres de l’Institut de recherche pour le développement et de l’Institut de recherche clinique du Bénin. Les antivenins ont été fournis gracieusement par Inosan Biopharma que nous remercions.

## Contributions des auteurs et autrices

SL : conception de l’étude, rédaction du protocole, coordination de l’étude, vérification et validation des données, analyse des données, rédaction du manuscrit

NS, NT, SAO, EG : recueil des données, correction du manuscrit

EAL : révision et validation du protocole, correction du manuscrit

AM : promoteur de l’étude, révision et validation du protocole, correction du manuscrit

JPC : conception de l’étude, acquisition du financement, validation du protocole, validation de l’analyse, révision et correction du manuscrit

## Déclaration de liens d’intérêts

Aucun lien d’intérêt n’a été déclaré.
