# Supplementary material for: Evaluation of the Efficacy and Tolerance of Tranexamic Acid Combined with InoserpTM PAN-AFRICA Antivenom in the Treatment of Hemorrhagic Syndrome during Envenomation by Echis ocellatus in Benin
Source: Med Trop Sante Int. 2026 Mar 15;6(1):mtsi.v6i1.2026.745. [Article in French] doi: 10.48327/mtsi.v6i1.2026.745 (PMC13216910; doi:10.48327/mtsi.v6i1.2026.745)
Supplement: Related texts

# Research article: Evaluation of the Efficacy and Tolerance of Tranexamic Acid Combined with Inoserp^TM^ PAN-AFRICA Antivenom in the Treatment of Hemorrhagic Syndrome during Envenomation by *Echis ocellatus* in Benin

## Introduction

In sub-Saharan Africa, snakebites are a neglected public health problem. More than 300,000 envenomations are treated in health centers each year [7]. In the Sudanese savanna, which includes northern Benin, a hemorrhagic syndrome contributes significantly to the mortality and morbidity associated with these envenomations. In this region, *Echis ocellatus* (West African Carpet Viper), a small viperid with powerful venom, is the species most often implicated. The bite of *E. ocellatus* is characterized by persistent local bleeding. In some cases, systemic hemorrhagic manifestations may occur, including blister, mucosal bleeding, gastrointestinal hemorrhage, and cerebral or meningeal hemorrhage. These complications can lead to death [39]. This bleeding results from a complex pathophysiology involving vascular destruction, platelet damage, and consumption coagulopathy. Consumption coagulopathy is mainly due to the presence of prothrombin activators, which convert prothrombin to thrombin. This results in the virtual disappearance of fibrinogen [16,34]. The treatment for ophidian envenomation, and consequently the hemorrhagic syndrome induced by the venom, relies on antivenom. Various studies have demonstrated its efficacy in treating bleeding and consumption coagulopathy [10,11,24]. However, persistent or recurrent bleeding has been reported in some patients during treatment. The current recommendation is to administer an additional dose of antivenom. In addition to increasing the risk of adverse effects [21], this approach increases the cost of treatment, which is generally borne by the patient. In Benin, the median cost of the antivenom required for a snakebite exceeds the average monthly income per capita [38]. Therefore, it is necessary to evaluate the benefits of less expensive treatments. Tranexamic acid is one such treatment, and is frequently used in health centers treating snake-bites in sub-Saharan Africa. It is an inexpensive antifibrinolytic that inhibits plasmin formation by blocking plasminogen’s lysine binding sites. However, little clinical data is available on its use in treating snakebite envenomation.

This study aimed to evaluate the efficacy and tolerance of combining tranexamic acid and antivenom to treat hemorrhagic syndrome and coagulopathy associated with *E. ocellatus* envenomation in Benin.

## Materials and methods

This phase III randomized controlled clinical trial was conducted at the Saint Jean de Dieu Hospital in Tanguiéta, northwestern Benin, from May 30 to October 29, 2023. Patients presenting with an active bleeding snake bite were included in the study.

Exclusion criteria included refusal to participate in the study, bites older than four days, administration of antivenom prior to arrival at the hospital, and the presence of a contraindication to tranexamic acid (e.g., arterial or venous thrombosis, severe renal failure with creatinine clearance less than 30 ml/min, current or reported history of seizures or epilepsy, pregnancy in the first trimester or breastfeeding, or known hypersensitivity to tranexamic acid).

Other exclusion criteria included creatinine clearance of less than 30 ml/min based on admission laboratory tests or the patient’s decision to withdraw from the study.

Patients who were not included received the standard treatment recommended by the Benin Ministry of Health.

Upon admission (H0), included patients were randomized into two groups: one receiving Inoserp^TM^ PAN-AFRICA and tranexamic acid (SAV + AT group), and one receiving only Inoserp^TM^ PAN-AFRICA (SAV group). Inclusion of a patient resulted in the creation of a data collection form on the EasyMedStat website (version 3.27; www.easy-medstat.com). The site automatically generated an identification number for each record: patients with an even number were included in the SAV + AT group, and patients with an odd number were included in the SAV group. However, it appeared that the generated numbers were systematically odd. Therefore, the inclusion criteria were reversed on August 16, after 13 patients had already been included. An even number was assigned to the SAV group, and an odd number was assigned to the SAV + AT group. Despite this change in the recruitment rule, the following three patients were assigned to the SAV group. Therefore, it was decided that starting on August 30, new patients would be systematically admitted to the SAV + AT group to balance the numbers, with nine new patients receiving SAV and AT.

Inoserp^TM^ PAN-AFRICA (Inosan Biopharma, Madrid, Spain) is a lyophilized, polyvalent antivenom (batches 2IT02001 and 3IT01001). The antivenom was administered according to the protocol recommended by the African Society of Venimology [8]. Each vial must be reconstituted with 10 mL of injectable saline solution. It neutralizes at least 250 LD_50_ of *E. ocellatus* venom. The recommended route of administration is a slow, direct intravenous injection over a minimum of five minutes. The initial dosage is two vials. A new clinical assessment is performed two hours (H2), four hours (H4), six hours (H6), eight hours (H8), 12 hours (H12), 24 hours (H24), 48 hours (H48), and 72 hours (H72) after the initial administration of the antivenom. Two vials of antivenom are administered again if bleeding persists or recurs. A final assessment was carried out on the day of discharge, regardless of the length of hospitalization.

Tranexamic acid (Exacyl, Kwality Pharmaceuticals Ltd., Amritsar, India, batch N-17207) is administered according to the following protocol: a loading dose of two 500-mg vials in a 250-mL infusion of 5% glucose over 30 minutes, followed by a maintenance dose of two 500-mg vials in a 500-mL infusion of 0.9% NaCl over eight hours intravenously. A maintenance dose was not administered if creatinine level was above 250 µmol/L upon admission. For children (patients under 18 years of age), the loading and maintenance doses were 10 mg/kg (maximum 1 g). Discontinuation of the tranexamic acid infusion was to result from the onset of visual disturbances, anuria, convulsions, or thrombosis. For patients in the SAV group, the following infusion was planned: 250 mL of 5% glucose over 30 minutes, followed by 500 mL of 0.9% NaCl over eight hours.

A standardized data collection form was completed for each patient including the following characteristics: gender, age, time between bite and admission, snake identification, edema and hemorrhage grade (Table I), presence of necrosis or other clinical signs, biological results, and treatment (number of antivenom and tranexamic acid vials administered, and the date and time of administration). Significant edema was defined as a grade of edema ≥ 2. Systemic bleeding was defined as a hemorrhage grade ≥ 2. The snakes responsible for the bites reported to the hospital were identified by a herpetologist. In this region of Benin, *E. ocellatus* is the only species responsible for envenomation with hemorrhagic syndrome. Therefore, even if the snake could not be brought in, we considered the bite to be from *E. ocellatus* if bleeding was detected upon admission. This data was entered into a secure online database on the EasyMedStat website.

The patient was monitored for one hour and during the specified visits above to check for signs of intolerance. Any symptoms or signs that appeared after the administration or re-administration of Inoserp^TM^ PAN-AFRICA or tranexamic acid that suggested possible intolerance were considered adverse events.

Upon admission, after inclusion, and before the administration of the antivenom, a venous whole blood sample was drawn to carry out a whole blood clotting test (WBCT), perform a complete blood count, and measure plasma creatinine.

The WBCT was performed according to the method described by Benjamin *et al*. [5]. A few milliliters of venous whole blood were placed in a clean glass tube and kept upright at room temperature. The WBCT was read at three-time points: 20, 30, and 60 minutes. The quality of the clot was assessed at each time point. A normal clot remains fixed when the tube is horizontal (grade 0). If the blood remains liquid or if a small clot forms that breaks down when the tube is handled, it is grade 1. The absence of a clot is grade 2. The WBCT is considered abnormal if clots are classified as 1 or 2 at any reading time. After the first antivenom dose was administered, samples were taken from patients at H2, H4, H6, H8, H12, H24, H48, H72, and on discharge day. A WBCT was performed on these samples, an INR was performed at H6 and H12, and a plasma creatinine assay was performed at H24 and H72. Adding INR to WBCT aligns with recent recommendations for defining endpoints in clinical trials on snakebites. It is recommended that this parameter be measured at H6 and H12 to evaluate the efficacy of antivenom in cases of envenomation with coagulopathy [2].

**Table I:** Criteria for grading edema and hemorrhage

Grade / Grade	œdème / Edema	Hémorragie / Hemorrhage
0	Absence / Absence	Absence / Absence
1	N’excédant pas une articulation / Not exceeding one joint	Persistance d’un saignement au site de morsure pendant plus de 30 minutes / Persistent bleeding at the bite site for more than 30 minutes
2	Atteignant deux articulations / Affecting two joints	Saignement des muqueuses ou des cicatrices récentes / Bleeding from mucous membranes or recent scars
3	Touchant l’ensemble du membre sans dépasser la racine / Affecting the entire limb without exceeding the root	Saignement sous-cutané à distance de la morsure ou des cicatrices anciennes / Subcutaneous bleeding away from the bite or old scars
4	Dépassant la racine / Exceeding the root	Externalisation d’un saignement interne ou saignement du système nerveux cérébral / Externalization of internal bleeding or bleeding from the cerebral nervous system

The primary endpoint was the absence of persistent or recurrent bleeding. Persistent bleeding is defined as bleeding that is still present at H2. Recurrent bleeding is defined as bleeding that is absent at H2 but present at least once during subsequent evaluations during follow-up.

The secondary endpoints were the recurrence of coagulopathy, defined as a WBCT returning to abnormal levels after correction at least once during follow-up, and the total number of vials of antivenom per patient. Other secondary endpoints included the presence of adverse effects and creatinine values at H24 and H72.

Statistical analysis was performed using GraphPad PRISM 9.5.0 (GraphPad Prism Inc., La Jolla, CA, USA). Qualitative variables were expressed as absolute values and percentages, and quantitative variables as medians, accompanied by 25% and 75% quartiles. The normality of quantitative variables was verified using the Shapiro-Wilk test. Due to the small sample size, we used the nonparametric Mann-Whitney test to compare the medians of quantitative variables and Fisher’s exact test to compare percentages. The objective of the study was to demonstrate that the combination of antivenom and tranexamic acid was more effective than antivenom alone. If the two strategies have similar efficacy, systematic addition would not be appropriate. Fisher’s exact test was therefore performed unilaterally to compare the groups on the outcome measures. The statistical difference in results was considered significant when p < 0.05.

This clinical trial was approved by the National Health Research Ethics Committee (CNERS) of Benin (Ethical Opinion No. 101/MS/DC/SGM/CNERS/SA of July 25, 2022). All participants (or, where applicable, their parents or guardians for minors) signed a written consent form after receiving clear, appropriate, and honest information.

## Results

Twenty-five patients were included: 12 in the SAV + AT group and 13 in the SAV group.

The epidemiological, clinical, and biological data at admission are presented in Table II. No statistically significant differences were found for these different variables. Two patients arrived just before the 4-day deadline. No skin necrosis or neurological manifestations were noted. The snake was reported in only one patient and was identified as an *E. ocellatus*.

Figure 1 shows the number of patients whose bleeding was corrected over time. Five patients (38%) in the SAV group and six patients (50%) in the SAV + AT group had persistent or recurrent bleeding (p = 0.837). There were four patients with persistent bleeding in each group (p = 0.714). All patients experienced resolution of persistent bleeding at H8 or later. Recurrent bleeding was noted in two patients in the SAV + AT group at H48 and H72, as well as in one patient in the SAV group after follow-up on day 4 (p = 0.904).

**Table II:** Description of patient characteristics at admission

	Groupe SAV + ATN = 12 / SAV + AT group = 12	Groupe SAV = 13 / SAV group = 12	>p-Value / p-Value
Sex-ratio homme/femme / Male/female sex ratio	8/4	10/3	0,673
Âge moyen (années) / Average age (years)	23 (± 11,0)	29 (± 11)	0,207
Délai moyen morsure/admission (heures) / Average time between bite and admission (hours)	47,2 (± 31,4)	32,6 (± 28,8)	0,236
Œdème important / Significant edema	11 (92 %)	9 (69 %)	0,322
Saignement systémique / Systemic bleeding	12 (100 %)	12 (92 %)	>0,999
TCTS anormal / Abnormal WBCT	12 (100 %)	13 (100 %)	>0,999
Hémoglobine (g/dl) / Hemoglobin (g/dL)	10,8 (± 4,2)	12,0 (± 3,7)	0,457
Plaquettes (G/l) / Platelets (G/L)	216 (± 95)	180 (± 56,6)	0,258
Créatinine (µmol/l) / Creatinine (µmol/L)	84 (± 25)	93 (± 23)	0,383

TCTS : test de coagulation sur tube sec / WBCT: Whole blood clotting test

**Figure 1:** Time-dependent correction of bleeding

Figure 2 shows the number of patients whose WBCT corrected over time. Six patients (50%) experienced recurrent coagulopathy in the SAV + AT group, compared to five patients (38%) in the SAV group (p = 0.837).

**Figure 2:** Time-dependent correction of whole blood clotting test

There was no significant difference between the two groups for INR measured at H6 (p = 0.978) and H12 (p = 0.663) (Fig. 3).

**Figure 3:** INR at the 6^th^ hour and at the 12^th^ hour after the first administration of antivenom

There was no significant difference between the two groups in the median total number of vials of antivenom administered per patient (p = 0.548) (Fig. 4).

**Figure 4:** Total number of antivenom vials per patient

No deaths were observed during the study. In the SAV + AT group, pruritus occurred in two patients, generalized urticaria in one patient, dizziness in one patient, and malaise in one patient. In the SAV group, dizziness occurred in two patients, and malaise occurred in one patient. No other adverse effects were reported, including hypotension, visual disturbances, convulsions, and thrombosis. There was no pathological increase in serum creatinine in the SAV + AT group, and no significant difference was observed in the SAV group for this variable at 24 hours (79.27 ± 17.71 µmol/L *versus* 86.36 ± 18.47 µmol/L, p = 0.339) or at 72 hours (83.16 ± 20.86 µmol/L versus 83.1 ± 14.65 µmol/L, p = 0.993).

## Discussion

To our knowledge, no studies have examined the combination of tranexamic acid and antivenom. Two prospective studies evaluated the benefits of tranexamic acid when administered alongside antivenom. The first study, conducted in Iraq, found no significant difference in mortality between patients who bled after being bitten and were treated with tranexamic acid (30% of 20 patients) and those who were not treated with tranexamic acid (48% of 23 patients) [1]. There were no endpoints related to bleeding or coagulopathy. The other study, conducted in Nepal on patients bitten by *Ahaetulla nasuta* (a potentially hemorrhagic Colubridae species), showed benefits in terms of INR correction on days 5 and 6, as well as shorter hospital stays [32]. The standard treatment for these patients was a combination of fresh frozen plasma and vitamin K.

There are several possible explanations for the persistence and recurrence of bleeding. Apart from an absence or delay in the regeneration of clotting factors — which is not corroborated by hepatic insufficiency [23,24,30,39] — the persistence or recurrence of the hemorrhagic syndrome could be attributed to two factors. The first is fibrinolysis secondary to toxigenic activation of clotting. The second is the release of residual venom from a sequestration site beyond the reach of the antivenom [9,19,20,28,30,33,35]. The combination of antivenom and tranexamic acid addresses the two aforementioned causes of bleeding. First, the half-life of the antivenom’s F(ab’)_2_ antibody fragments in the plasma compartment is around 50 hours, which should allow the circulating venom to be neutralized for several days [4,31]. Second, tranexamic acid was intended to control hyperfibrinolysis, especially if it persisted after the venom was eliminated.

However, in this clinical trial on the treatment of hemorrhagic syndrome and coagulopathy associated with *E. ocellatus* bites, the combination of antivenom and tranexamic acid was ineffective. In some patients, antivenom does not seem to prevent persistent or recurrent bleeding. This phenomenon is not limited to Inoserp^TM^ Pan-Africa; it has also been observed with other antivenoms, including Antivipmyn™ Africa (Bioclon, Mexico City, Mexico) [10], EchiTAb Plus-ICP (Clodomiro Picado Institute, Vázquez de Coronado, Costa Rica) [12,37], and EchiTAb G (MicroPharm Ltd., Newcastle Emlyn, UK) [3,12]. These findings suggest that recurrent coagulopathy is independent of the antivenom used and appears to be related to the release of venom that is not neutralized by the antivenom [33]. The mechanisms underlying this secondary release remain unknown.

There are several hypotheses that could explain the ineffectiveness of tranexamic acid in our study. First, hyperfibrinolysis is not consistent during envenomation by *E. ocellatus*. Of the patients studied using viscoelastometry in Tanguiéta, only 26% presented with hyperfibrinolysis [19]. In severely traumatized patients, tranexamic acid has only been shown to be useful in correcting hemostasis disorders in patients with hyperfibrinolysis [26]. Ideally, tranexamic acid would be used only in patients with this condition, but it is usually impossible to make this diagnosis routinely. An alternative would be to target envenomations in which hyperfibrinolysis is prevalent and the primary hemostasis disorder. The venom of *Ahaetulla prasina*, a species closely related to *Ahaetulla nasuta*, has not been studied to our knowledge. Its venom is predominantly composed of type III metalloproteinases and exhibits fibrinogenolytic activity [25]. Therefore, it is possible that the bleeding observed during *A. nasuta* envenomation is due to hyperfibrinolysis. This would explain the effectiveness of INR correction in a study conducted in Nepal on patients envenomated by *A. nasuta* [32]. Other snake species are responsible for envenomations dominated by this pathophysiological mechanism. For example, *Hypnale hypnale*, a viperid found in Sri Lanka and southern India, has venom that causes coagulopathy marked by isolated fibrinogenolysis [29]. Therefore, the use of tranexamic acid could be studied in envenomations caused by this species.

The main hypothesis explaining the failure of tranexamic acid is the significant decrease in fibrinogen observed during envenomation by species of the genus *Echis* [24]. The intense consumption of clotting factors, particularly fibrinogen, leads to their near disappearance. Antivenom alone does not restore these factors, and their hepatic synthesis requires an unavoidable delay. This results in a very small clot for several days despite the antivenom [19]. Hyperfibrinolysis can destabilize this clot, but correcting it is insufficient to ensure effective hemostasis and stop bleeding. In a porcine model of severe liver trauma, it has been demonstrated that, in cases of profound hypofibrinogenemia, tranexamic acid alone cannot restore hemostasis, and the addition of fibrinogen concentrate is essential [42]. Combining this with a transfusion of clotting factors may be beneficial to correct this deficiency more quickly. However, fibrinogen concentrate is ruled out due to its cost and lack of availability in peripheral health centers, which treat the majority of patients. An alternative is a transfusion of fresh frozen plasma, which contains at least 2.5 g/L of fibrinogen [14] and is recommended for disseminated intravascular coagulopathy with hemorrhagic manifestations [22]. Additionally, it is inexpensive, making it affordable for most patients. A clinical trial conducted in Australia showed that the INR was corrected more quickly in patients receiving fresh frozen plasma in addition to antivenom [17]. Another benefit of adding fresh frozen plasma, observed in Sri Lanka and India, is reducing the number of antivenom vials needed, thus reducing the cost of treatment [15, 18]. However, plasma transfusion carries a risk of transmitting bloodborne agents, such as HIV, HBV, and HCV, in Africa [40,41].

On the other hand, we have found that tranexamic acid is well tolerated in cases of snakebite. In particular, no thrombotic or convulsive complications have occurred. In theory, the inhibition of fibrinolysis could lead to clot formation due to the intense activation of coagulation by the venom. However, the consumption of clotting factors results in a pronounced hypocoagulant phenotype that safeguards patients against this thrombotic risk. Previous studies on ophidian envenomation also reported excellent tolerance [1,32]. Tranexamic acid is generally well tolerated, especially at the typical dosage of 1 to 2 g/day. According to several meta-analyses, thromboembolic events do not appear to increase significantly [27,36]. Seizures are a rare, dose-dependent risk, especially at high doses or during cardiac surgery [6,26].

Several limitations of this study should be noted. First, this study has a modest sample size; however, a larger number of patients would probably not have demonstrated the efficacy of tranexamic acid. In particular, most bleeding involved only the mucous membranes, and there were few severe cases. Similarly, the absence of adverse events may be due to the sample size. Changes in the recruitment rules in both groups during the study led to an absence of true randomization. However, this is unlikely to have impacted the study because there is no reason to believe that snake venoms change over such a short period of time. Additionally, we used the same batch of drugs throughout the study. Since most bites occur between May and October, enrollment ended at the end of October. Analysis of the data led us to decide not to continue the study during the following bite season. Excess plasmin generated during hyperfibrinolysis consumes fibrinogen [13]. It would have been interesting to measure this parameter; however, we opted for tests that are either routinely available, such as WBCT, or recommended in antivenom clinical trials, such as INR.

## Conclusion

Adding tranexamic acid to antivenom does not appear to benefit the treatment of bleeding associated with *E. ocellatus* bites in Benin. However, this inexpensive treatment warrants further study in countries where envenomation is characterized by frequent and predominant hyperfibrinolysis, such as with *H. hypnale* bites in the Indian subcontinent.

## Acknowledgments

We would like to thank the staff at the Saint Jean de Dieu Hospital in Tanguiéta for their hospitality and assistance during this study.

## Funding

This study was funded by the Research Institute for Development and the Clinical Research Institute of Benin. The antivenoms were provided free of charge by Inosan Biopharma, for which we are grateful.

## Authors’s contributions

SL: Study design, protocol drafting, study coordination, data verification and validation, and data analysis.

NS, NT, SAO, EG: Data collection and manuscript editing.

EAL: protocol review and validation, manuscript correction

AM: Study sponsor, protocol review and validation, and manuscript correction.

JPC: study design, funding acquisition, protocol validation, analysis validation, manuscript revision, and correction.

## Conflicts of interest

The authors declare no conflicts of interest.
